# The associations of increased cerebral small vessel disease with cognitive impairment in neurosyphilis presenting with ischemic stroke

**DOI:** 10.1002/brb3.2187

**Published:** 2021-05-16

**Authors:** Lei Xiang, Tao Zhang, Biao Zhang, Chao Zhang, Shuping Hou, Wei Yue

**Affiliations:** ^1^ Tianjin Key Laboratory of Cerebrovascular and Neurodegenerative Diseases Department of Neurology Tianjin Huanhu Hospital Tianjin China; ^2^ Department of Intensive Care Unit Tianjin Huanhu Hospital Tianjin China; ^3^ Department of Clinical Laboratory Tianjin Huanhu Hospital Tianjin China; ^4^ Department of Dermatovenereology Tianjin Medical University General Hospital Tianjin China

**Keywords:** cerebral small vessel disease, cognitive impairment, ischemic stroke, microbleeds, neurosyphilis, white matter hyperintensity

## Abstract

**Background and Purpose:**

Ischemic stroke is a common clinical feature of neurosyphilis, but its accompanying cognitive decline is often overlooked. The mechanisms of cognitive impairment in neurosyphilis presenting with ischemic stroke are not fully understood. Cerebral small vessel disease (CSVD) was recently shown to predict post‐stroke cognitive decline. Therefore, this study aims to validate the correlation between CSVD and cognitive impairment in neurosyphilis presenting with ischemic stroke.

**Methods:**

We enrolled 179 neurosyphilis patients diagnosed as acute ischemic stroke and performed a 12‐month cognitive assessment follow‐up. CSVD burden was evaluated by neuroimaging markers, including white matter hyperintensities (WMHs), lacunes, cerebral microbleeds (CMBs), and perivascular spaces (PVS). We performed multivariate logistic regression analysis to determine the association between cognitive decline and total CSVD burden score in neurosyphilis patients.

**Results:**

The neurosyphilis participants had a significantly higher total CSVD score and lower cognitive function score compared with the syphilis‐uninfected patients. Acute cognitive impairment was associated with total CSVD score, extensive microbleeds, and Grade 3 WMHs. After 12‐month follow‐up, the poor prognosis of post‐stroke cognitive impairment was associated with a higher burden of CSVD and extensive microbleeds.

**Conclusions:**

Cerebral small vessel disease loads in neurosyphilis patients presenting with ischemic stroke are independently associated with acute cognitive impairment and have a prospective value for post‐stroke cognitive outcomes.

## Graphical abstract

Cerebral small vessel disease in neurosyphilis patients presenting with ischemic stroke is independently associated with acute cognitive impairment and have a prospective value for post‐stroke cognitive outcomes.

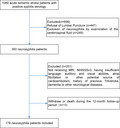

## 1 INTRODUCTION

The incidence of syphilis remains on the rise in China, for which statistics are available (Chen et al., 2007; Tang et al., 2017). The misdiagnosis rate of neurosyphilis was high because the clinical and neuroimaging presentations are extremely diverse. Progressive cognitive decline is a typical manifestation of late neurosyphilis, known as general paresis. However, mild cognitive decline has been observed in meningovascular syphilis, the most common form of neurosyphilis, of which stroke is a primary symptom (Liu et al., 2012). Syphilis‐related cognitive decline is very common but has heterogenous mechanisms that are not fully understood, particularly in neurosyphilis patients presenting with ischemic stroke.

Cerebral small vessel disease (CSVD) is a consequence of pathological alterations of the brain's small arteries, which is one of the major causes of cognitive decline (Wardlaw et al., 2013). The markers of CSVD on magnetic resonance imaging (MRI) are white matter hyperintensities (WMHs), cerebral microbleeds (CMBs), asymptomatic lacunar infarctions (ALIs), and enlarged perivascular spaces (EPVS). All these markers are individually associated with cognitive decline, and the combined effect of those has also been linked to cognitive decline and poor prognosis of stroke (Huijts et al., 2013; Lau et al., 2017; Uiterwijk et al., 2016). The few reported studies on the correlation between CSVD and dementia of patients with neurosyphilis have mainly been single case reports, usually consisted of nonspecific findings in patients with meningovascular neurosyphilis (Brinar & Habek, 2006). Our previous study suggested that ischemic stroke individuals with positive syphilis serology were more prevalent in intracranial artery stenosis (Xiang et al., 2021), and small vessels were also involved in these individuals. It is possible that neurosyphilis is associated with an increased risk of CSVD because the damage in cerebral small vessels is one of the typical pathological features of neurosyphilis. However, whether CSVD is more common in neurosyphilis‐related ischemic stroke patients compared with syphilis‐negative individuals remains unclear. No data are available on the cognitive consequences of the CSVD burden in neurosyphilis presenting with ischemic stroke.

Since the routine screening of syphilis serology in stroke patients in our stroke center, the diagnostic rate of neurosyphilis presenting as acute ischemic stroke has been increasing, which led us to undertake this study. We aimed to determine whether neurosyphilis was associated with increased cerebral CSVD burden in patients with minor ischemic stroke. We also sought to confirm the correlation between CSVD burden and post‐stroke cognitive decline and estimate its predictive value for post‐stroke cognitive recovery in neurosyphilis patients presenting as acute minor ischemic stroke.

## 2 METHODS

### 2.1 Study sample

Patients with first‐ever ischemic stroke admitted to the Tianjin Huanhu hospital from March 2013 to September 2018 were consecutively screened for the following study entry criteria: receiving MRI on admission, the NIH Stroke Scale (NHISS) < 3, having sufficient language, auditory, and visual abilities to allow the assessments. The diagnosis of acute ischemic stroke was defined as meeting the definition of acute ischemic stroke according to the American Heart Association/American Stroke Association World Health Organization (Sacco et al., 2013). Patients with atrial fibrillation and other potential sources of cardioembolism, history of previous TIA/stroke, dementia, or other neurological diseases were excluded.

All the stroke patients were screened for syphilis serology. When serology proves positive, all patients should undergo cerebrospinal fluid (CSF) examination. Patients who had positive syphilis serologies and met the following criteria were allocated to neurosyphilis (Timmermans & Carr, 2004; “1998 Guidelines for Treatment of Sexually Transmitted Diseases. Centers for Disease Control and Prevention,” 1998). 1) Positive cerebrospinal fluid VDRL (Venereal Disease Research Laboratory) or 2) Positive cerebrospinal fluid FTA‐abs (fluorescent treponemal antibody absorption test), with either abnormal CSF cell count (polymorphonuclear leucocytes and/or lymphocytes >5/ml), or CSF protein>0.45 g/L, or IgG index>0.6. We also enrolled a group of control subjects who matched patients (1:1) by sex and age (±3 years) and fulfilled the inclusion and exclusion criteria reported above during the same study period, except they had negative syphilis serology. During admission, demographic data, past medical history, and atherosclerotic risk factors were registered. TOAST classification in our study included large artery atherosclerosis (LAA), small vessel occlusion (SVO), stroke of other determined etiologies (SOE), and stroke of undetermined etiologies (SUE), because cardioembolism (CE) were excluded in our samples. Written informed consent was obtained from every patient, and this study protocol was approved by the Clinical Research Ethics Committee of Tianjin Huanhu Hospital and performed in accordance with the ethical standards in the Declaration of Helsinki.

### 2.2 Brain MRI and definition of CSVDs

All patients were scanned using a 3.0T GE scanner within 7 days after developing neurological symptoms. MRI sequences included diffusion‐weighted imaging, T2*‐weighted gradient‐recalled echo proton‐density weighted, T1‐ and T2‐weighted and fluid‐attenuated inversion recovery. All MR images were reviewed by a senior neurologist blinded to all other data. The neuroimaging markers of SVD were all defined outside the area of acute cerebral infarction.

Asymptomatic lacunar infarctions (ALIs) were considered as circular or cavitary lesions, >3 and <20 mm in diameter on T2 and fluid‐attenuated inversion recovery (FLAIR) and no increased signal on diffusion‐weighted imaging (DWI), with no relevant history of neurological signs or symptoms (Wardlaw et al., 2013). The presence of CMBs/Microbleeds was indicated by hypodense foci up to 10 mm on gradient echo sequences (GRE) images and was differentiated from microbleed mimics based on current guidelines (Greenberg et al., 2009). The location and number of microbleeds were scored according to the Microbleed Anatomical Rating Scale (Gregoire et al., 2009), and microbleed‐burden graded as absent, 1, 2–4, and ≥5. Periventricular and subcortical WMH were assessed, respectively, according to the Fazekas scale ranging from 0 to 3 (periventricular WMH: 0 = absent or small triangular foci surrounding the frontal horns, 1 = caps or thin line surrounding the anterior and posterior horns, 2 = extensive patchy and their early confluent stages, 3 = confluent, completely surrounding lateral ventricles; subcortical WMH: 0 = absent, 1 = punctate foci, 2 = beginning confluence of foci, and 3 = large confluent areas) (Fazekas et al., 1987). The severity of enlarged PVSs in the basal ganglia was graded according to the number of spaces based on a previously validated scale (Potter et al., 2015). The burden of enlarged PVSs was stratified into three groups: <10, 10–20, and >20, and only BG‐PVS were used in the total SVD score.

The total SVD score was calculated to assess the overall burden of SVD according to the STRIVE guideline (Staals et al., 2014). One point is allocated to each of the following parameters: presence of lacunes; existence of cerebral microbleeds (CMBs); moderate–severe (>10) basal ganglia (BG) perivascular spaces (PVS); and severe periventricular or moderate–severe deep white matter hyperintensities (WMHs).

### 2.3 Assessment of cognitive function

Participants underwent a cognitive function assessment by one trained neuropsychologist using the Montreal Cognitive Assessment (MoCA) tool. Patients with <12 years of education were assigned 1 additional point on their MoCA score. The acute MoCA was collected immediately after admission within 7 days after acute stroke. All participants were invited to undergo additional MoCA at 12 months (“12 months MoCA”). A MoCA score <26 was considered cognitive impairment (Pendlebury et al., 2010). Patients with an acute MoCA score <26 were divided into two groups according to 12 months MoCA. “Reverters” were defined as patients who demonstrated an improvement of ≥2 points at 12 months, while patients who did not show this improvement were defined as “nonreverters.” These thresholds are defined based on previously published work (Sivakumar et al., 2014).

### 2.4 Statistical analyses

Continuous variables were presented as mean and standard deviation (mean ± *SD*), whereas categorical variables were presented as percentages. We compared the demographic, clinical, and imaging characteristics of patients using *t* test for normally distributed continuous variables, Kruskal–Wallis test for skewed continuous variables, and chi‐square test for categorical variables. Difference between variables in Table 3 were examined by independent *t* test or Mann–Whitney *U* test. Logistic regression models analysis was utilized to determine the relationships of cognitive function (including acute cognitive impairment and 12‐month cognitive evolution) with four individual CSVD markers as well as the total SVD score. All models were adjusted for potential confounders known to affect cognitive function, including age, gender, years of education, admission NIHSS, obesity, hyperlipidemia, history of hypertension, diabetes, coronary heart disease, drinking, and smoking. A *p* value of < .05 was considered significant. All analyses were performed using SPSS version 19.0 (SPSS Inc).

## 3 RESULTS

### 3.1 The clinical and neuroimaging characteristics of the study population

**FIGURE 1:**
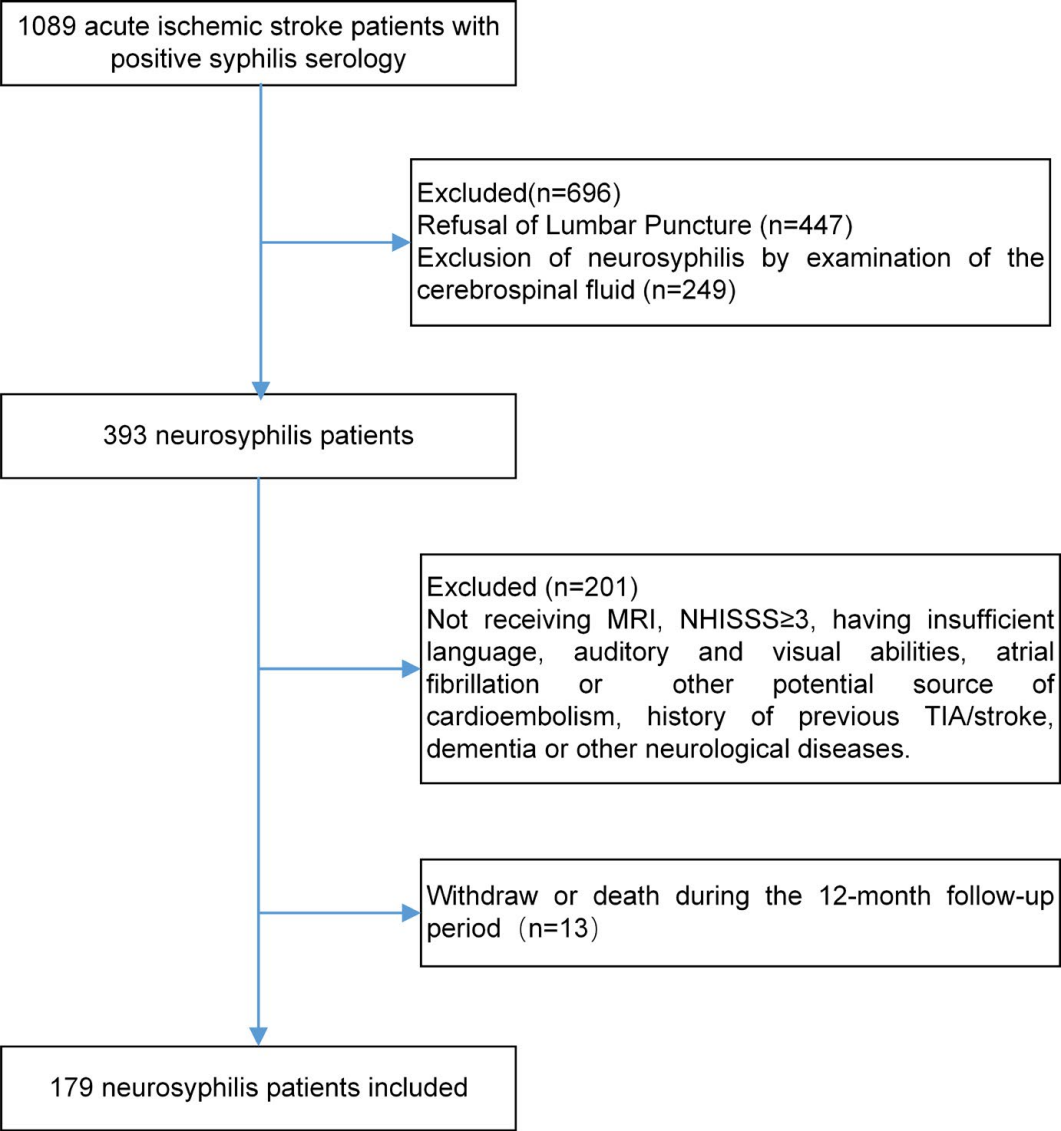
Flowchart of neurosyphilis patients recruited into the study

A flowchart of the neurosyphilis patients' recruitment is shown in Figure 1. Among 1,089 consecutive patients diagnosed with acute ischemic stroke with positive syphilis serology at Tianjin Huanhu hospital from March 1, 2013, to September 30, 2018, only 393 patients were diagnosed with neurosyphilis. Eventually, 179 neurosyphilis patients were included in the final analyses according to inclusion and exclusion criteria, after excluding the participants who subsequently dropped out or died at 12 months after recruitment. Control group matched neurosyphilis patients (1:1) and fulfilled the same admission and exclusion criteria during the same study period, except they had negative syphilis serology.

The baseline clinical and neuroimaging characteristics of the study participants are shown in Table 1. The neurosyphilis patients were more likely to be alcohol drinkers (52.5% vs. 30.7%; *p* < .001), while patients without syphilis were more likely to have hypertension (65.4% vs. 55.3%; *p* = .040). There were no differences in other vascular risk factors, including diabetes, hyperlipidemia, smoking status, and coronary heart disease between the two groups. The neurosyphilis participants had a lower acute MoCA score than the patients without syphilis (22.0 ± 3.0 vs. 26.0 ± 1.3; *p* < .001), but there was no difference in years of education. There were no differences in the proportion of patients with enlarged basal ganglia PVSs and periventricular WMHs between the two groups. However, patients with neurosyphilis had a greater burden of ALIs (39.7% vs. 16.8%, *p* < .001), high‐grade subcortical WMHs (Grade 2:26.3 vs. 18.4, *p* = .029; Grade 3:16.2 vs. 8.4, *p* = .035), and mixed location CMBs (21.8 vs. 12.3, *p* = .024). Consequently, the mean total CSVD score was significantly higher in the neurosyphilis cohort compared with the syphilis‐uninfected patients (1.9 ± 0.2 vs. 1.7 ± 0.3, *p* < .001).

**TABLE 1:** The baseline clinical and neuroimaging characteristics of study participants according to the presence of neurosyphilis

	Total *n* = 358		Neurosyphilis *n* = 179	Non‐syphilis *n* = 179	*p*
Characteristics					
Age (y)	61.6 ± 13.1		61.6 ± 13.3	61.7 ± 13.0	.911
Gender (male/female)	286/72		143/36	143/36	1.000
Education (y)	10.3 ± 2.5		10.1 ± 2.3	10.6 ± 2.4	.458
Drinking (*n*, %)	149 (41.6)		94 (52.5)	55 (30.7)	<.001
Smoking (*n*, %)	212 (59.2)		99 (55.3)	113 (63.1)	.162
Obesity (*n*, %)	149 (41.6)		76 (42.5)	73 (40.8)	.830
Diabetes (*n*, %)	111 (31.0)		56 (31.3)	55 (30.7)	.999
Hypertension (*n*, %)	216 (60.3)		99 (55.3)	117 (65.4)	.040
Hyperlipidemia (*n*, %)	202 (56.4)		104 (58.1)	98 (54.7)	.594
Coronary heart disease (*n*, %)	116 (32.4)		56 (31.3)	60 (33.5)	.735
Admission NHISS score	2.1 ± 0.6		2.0 ± 0.5	2.1 ± 0.7	.148
Acute MoCA score	24.8 ± 2.3		22.0 ± 3.0	26.0 ± 1.3	<.001
TOAST classification					
LAA (*n*, %)	88 (24.6)		46 (25.7)	42 (23.5)	.857
SVO (*n*, %)	187 (52.2)		89 (49.7)	98 (54.7)	.236
SOE or SUE (*n*, %)	83 (23.2)		44 (24.6)	39 (21.8)	.675
Imaging characteristics					
With ALIs (*n*, %)	101 (28.2)		71 (39.7)	30 (16.8)	<.001
With periventricular WMHs (*n*, %)					
Grade 1	82 (22.9)		45 (25.1)	37 (20.7)	.379
Grade 2	37 (10.3)		20 (11.2)	17 (9.5)	.729
Grade 3	12 (3.4)		8 (4.5)	4 (2.2)	.374
With subcortical WMHs (*n*, %)					
Grade 1	147 (41.1)		79 (44.1)	68 (38.0)	.283
Grade 2	80 (22.3)		47 (26.3)	33 (18.4)	.029
Grade 3	44 (12.3)		29 (16.2)	15 (8.4)	.035
With CMBs (*n*, %)	138 (38.5)	76 (42.5)		62 (34.6)	.158
1	61 (17.0)	32 (17.9)		29 (16.2)	.779
2–4	50 (14.0)	27 (15.1)		23 (12.8)	.648
≥5	27 (7.5)	17 (9.5)		10 (5.6)	.229
Strictly deep	36 (10.1)	16 (8.9)		20 (11.2)	.599
Strictly lobar	25 (7.0)	13 (7.3)		12 (6.7)	.999
Strictly infratentorial	16 (4.5)	8 (4.5)		8 (4.5)	1.000
Mixed location	61 (17.0)	39 (21.8)		22 (12.3)	.024
With enlarged basal ganglia PVSs (*n*, %)					
<10	230 (64.2)	116 (64.8)		114 (63.7)	.877
10–20	88 (24.6)	46 (25.7)		42 (23.5)	.576
>20	40 (11.2)	17 (9.5)		23 (12.8)	.539
Total CSVD score	1.8 ± 0.3	1.9 ± 0.2		1.7 ± 0.3	<.001

Note

Values are expressed as the mean ± *SD* for a normal distribution and as the absolute number (percentage) for categorical variables.

Abbreviations: ALIs, asymptomatic lacunar infarctions; CMBs, cerebral microbleeds; CSVD, cerebral small vessel disease; LAA, large artery atherosclerosis; MoCA, montreal cognitive assessment; NHISS, the NIH stroke scale; PVS, perivascular spaces; SOE, stroke of other determined etiologies; SUE, stroke of undetermined etiologies; SVO, small vessel occlusion; WMHs, white matter hyperintensities.

### 3.2 Association between the presence of CSVDs and acute post‐stroke cognitive impairment in neurosyphilis patients

Among the 179 neurosyphilis patients, 84 individuals had an admission MoCA score <26, which were considered acute post‐stroke cognitive impairment. To determine whether there is a correlation between high CSVD burden and acute post‐stroke cognitive impairment in neurosyphilis patients, we used logistic regression models adjusted for demographic, years of education, stroke risk factors, and admission NIHSS. The association between CSVDs and acute cognitive impairment in neurosyphilis patients is presented in Table 2. In univariate analysis, acute cognitive impairment was significantly associated with the increasing burden of microbleeds, especially those with ≥5 microbleeds (OR 3.03, 95% CI 1.06–8.62, *p* = .037, Table 2). In adjusted analyses, acute cognitive impairment remained associated with extensive microbleeds (OR 3.37, 95% CI 1.01–11.02, *p* = .041, Table 2), the associations with Grade 3 WMHs were enhanced (periventricular WMHs OR 3.30, 95% CI 2.20–15.01, *p* = .001; subcortical WMHs OR 3.11, 95% CI 1.02–9.51, *p* = .046; Table 2). When the four individual CSVD markers were combined into a “CSVD score,” the overall burden of CSVD was significantly correlated with acute cognitive impairment, and the correlation was strengthened after adjusting for covariates (OR 6.82, 95% CI 2.77–16.79, *p* < .001; Table 2).

**TABLE 2:** Unadjusted and adjusted model for imaging characteristics related to acute post‐stroke cognitive impairment in neurosyphilis patients

Variables	Univariate odds ratio (95% CI)	*p*	Adjusted model^*^ odds ratio (95% CI)	*p*
Total CSVD score	3.80 (1.80–8.01)	<.001	6.82 (2.77–16.79)	<.001
With ALIs (%)	1.04 (0.55–1.96)	.881	1.16 (0.60–2.25)	.641
With periventricular WMHs (%)				
Grade 1	1.77 (0.85–3.67)	.124	1.72 (0.83–3.60)	.144
Grade 2	1.92 (0.80–4.57)	.139	1.72 (0.69–4.31)	.242
Grade 3	2.87 (0.85–9.61)	.087	3.30 (2.20–15.01)	.001
With subcortical WMHs (%)				
Grade 1	0.84 (0.46–1.54)	.586	0.94 (0.50–1.78)	.871
Grade 2	1.79 (0.88–3.61)	.104	1.56 (0.71–3.46)	.266
Grade 3	2.57 (0.94–7.00)	.064	3.11 (1.02–9.51)	.046
With CMBs (%)	1.58 (0.31–2.08)	.087	2.35 (0.90–6.11)	.080
1	1.74 (0.74–4.07)	.199	2.39 (0.92–6.19)	.073
2–4	1.89 (0.55–6.46)	.306	2.91 (0.93–9.08)	.065
≥5	3.03 (1.06–8.62)	.037	3.37 (1.01–11.02)	.041
Strictly deep	3.91 (0.73–20.77)	.109	4.07 (0.73–22.71)	.101
Strictly lobar	2.34 (0.63–8.61)	.201	1.49 (0.38–5.85)	.563
Strictly infratentorial	2.55 (0.79–8.13)	.114	2.14 (0.65–7.03)	.207
Mixed location	1.99 (0.74–5.33)	.168	2.05 (0.72–5.82)	.178
With enlarged basal ganglia PVSs (%)				
<10	1.33 (0.92–1.92)	.119	1.42 (0.92–2.17)	.106
10–20	1.53 (0.51–4.60)	.451	3.68 (0.62–21.79)	.151
>20	1.22 (0.15–9.72)	.850	2.14 (0.19–23.61)	.535

Abbreviations: ALIs, asymptomatic lacunar infarctions; CMBs, cerebral microbleeds; CSVD, cerebral small vessel disease; MoCA, montreal cognitive assessment; PVS, perivascular spaces; WMHs, white matter hyperintensities.

^*^ Adjusted for age, gender, education, admission NIHSS, obesity, hyperlipidemia, history of hypertension, diabetes, coronary heart disease, drinking, smoking.

### 3.3 The evolution of cognitive function and its correlation with CSVD in neurosyphilis patients

We quantified cognitive impairment using MoCA performance and studied cognitive function evolution in 179 neurosyphilis patients. The MoCA was divided into seven cognitive domains, including visuospatial, naming, attention, language, abstract reasoning, recall, and orientation. Mean scores for each subtest are summarized in Table 3, used to analyze performance in specific cognitive domains. All 179 neurosyphilis patients participated in MoCA screening test at baseline within 7 days after stroke and 12 months after stroke. There was an improvement from the acute stage to 12 months in the total score (mean difference 2.00 points where the maximum achievable score is 30, *p* < .001; Table 3). Although most subtest scores remained stable over 12 months, visuo‐executive function (mean difference 0.75 points, *p* = .044) and delayed recall (mean difference 0.55 points, *p* = .045) subtest scores improved significantly at 12 months.

**TABLE 3:** Comparison of acute and 12 mo post‐stroke MoCA performance

	Comparison of means				
	Maximum achievable score	Acute post‐stroke MoCA Mean score (*SD*)	12 mo post‐stroke MoCA Mean score (*SD*)	Mean difference （95％ CI）	*p*
Total score	30	22.0 ± 3.0	24.0 ± 2.9	2.00 (0.95–3.04)	<.001
Visuo‐executive	5	3.20 ± 1.50	3.95 ± 1.23	0.75 (0.02–1.47)	.044
Naming	3	2.20 ± 0.83	2.30 ± 0.80	0.10 (−0.10–0.30)	.330
Attention	6	4.85 ± 1.08	5.00 ± 1.02	0.15 (−0.16–0.46)	.332
Language	3	2.20 ± 0.83	2.40 ± 0.75	0.20 (−0.08–0.48)	.161
Abstraction	2	1.35 ± 0.48	1.45 ± 0.51	0.10 (−0.04–0.24)	.163
Recall	5	3.75 ± 1.07	4.30 ± 0.80	0.55 (0.01–1.08)	.045
Orientation	6	4.55 ± 1.53	4.40 ± 1.84	0.15 (−0.53–0.83)	.651

Note

Values are expressed as the mean ± *SD*.

Abbreviations: MoCA, montreal cognitive assessment.

Based on the improvement/worsening of cognitive function by 12 months, neurosyphilis patients with acute cognitive impairment (*n* = 84; MoCA <26) were divided into two groups, reverters and nonreverters (Table 4). A comparison of demographic, clinical, and MRI characteristics was detailly listed in Table 4. There were no significant differences in demographic characteristics and prevalence of vascular risk factors, except that coronary heart disease was more common in nonreverters (*p* = .020). A comparison of CSVD characteristics showed that the prevalence of ALIs (57.1 vs. 32.7, *p* = .043) and extensive microbleeds (22.8 vs. 4.1, *p* = .014) was higher in nonreverters than reverters. There was a significant difference in the total burden of CSVD between nonreverters and reverters (2.4 ± 1.0 vs. 1.8 ± 0.6, *p* = .002).

**TABLE 4:** A comparison of baseline and imaging characteristics between reverters and nonreverters

	Reverters	Nonreverters	*p*
	*n* = 49	*n* = 35	
Baseline Characteristics			
Age (y)	55.8 ± 16.1	58.8 ± 13.8	.381
Gender (male/female)	36/13	28/7	.606
Education (y)	10.4 ± 3.3	9.9 ± 4.6	.130
Drinking (*n*, %)	27 (55.1)	18 (51.4)	.826
Smoking (*n*, %)	28 (57.1)	19 (54.3)	.827
Admission NHISS score (*n*, %)	1.88 ± 0.72	2.03 ± 0.17	.231
Obesity (*n*, %)	19 (38.8)	14 (40.0)	.998
Diabetes (*n*, %)	18 (36.7)	15 (42.9)	.653
Hypertension (*n*, %)	24 (49.0)	16 (45.7)	.827
Hyperlipidemia (*n*, %)	26 (53.1)	13 (37.1)	.186
Coronary heart disease (*n*, %)	7 (14.3)	13 (37.1)	.020
Admission NHISS score	1.9 ± 0.7	2.0 ± 0.1	.231
Imaging characteristics			
With ALIs (*n*, %)	16 (32.7)	20 (57.1)	.043
With periventricular WMHs (*n*, %)			
Grade 1	12 (24.5)	9 (25.7)	.999
Grade 2	6 (12.2)	5 (14.3)	.998
Grade 3	3 (6.1)	6 (17.1)	.154
With subcortical WMHs (*n*, %)			
Grade 1	17 (34.7)	16 (45.7)	.368
Grade 2	10 (20.4)	9 (25.7)	.605
Grade 3	5 (10.2)	4 (11.4)	.999
With CMBs (*n*, %)	17 (34.7)	22 (62.9)	.015
1	10 (20.4)	6 (17.1)	.784
2–4	5 (10.2)	8 (22.9)	.135
≥5	2 (4.1)	8 (22.8)	.014
Strictly deep	3 (6.1)	6 (17.1)	.154
Strictly lobar	2 (4.1)	5 (14.3)	.122
Strictly infratentorial	3 (6.1)	3 (8.6)	.690
Mixed location	9 (18.4)	8 (22.9)	.784
With enlarged basal ganglia PVSs (*n*, %)			
<10	26 (53.1)	17 (48.6)	.872
10–20	13 (26.5)	10 (28.6)	.917
>20	10 (20.4)	8 (22.9)	.938
Total CSVD score	1.8 ± 0.6	2.4 ± 1.0	.002

Note

Values are expressed as the mean ± *SD* for a normal distribution and as the absolute number (percentage) for categorical variables.

Abbreviations: ALIs, asymptomatic lacunar infarctions; CMBs, cerebral microbleeds; CSVD, cerebral small vessel disease; NHISS, the NIH Stroke Scale;PVS, perivascular spaces; WMHs, white matter hyperintensities.

To identify whether the higher burden of CSVD is associated with no improvement of cognitive impairment during the 12‐month follow‐up, we performed further multivariable binary logistic regression. In univariate analysis, the total CSVD score was independently associated with nonreverters (OR 2.31, 95% CI 1.03–5.20, *p* = .041, Table 5). After adjusting for covariates, nonreverters was also associated with extensive microbleeds (OR 4.78, 95% CI 1.18–19.28, *p* = .028, Table 5), a similar association was observed in total burden of CSVD (OR 2.56, 95% CI 1.06–6.18, *p* = .036, Table 5). Thus, the total CSVD score was a significant predictor of no improvement in cognitive impairment.

**TABLE 5:** Unadjusted and adjusted model for imaging characteristics related to no improvement of cognitive impairment during 12‐mo follow‐up

Variables	Univariate odds ratio (95% CI)	*p*	Adjusted model * odds ratio (95% CI)	*p*
Total CSVD score	2.31 (1.03–5.20)	.041	2.56 (1.06–6.18)	.036
With ALIs (%)	1.17 (0.49–2.82)	.711	1.15 (0.47–2.79)	.754
With periventricular WMHs (%)				
Grade 1	1.15 (0.43–3.07)	.772	1.21 (0.39–3.67)	.735
Grade 2	1.17 (0.34–3.93)	.799	1.12 (0.27–4.51)	.871
Grade 3	1.53 (0.47–4.97)	.472	3.14 (0.70–14.07)	.133
With subcortical WMHs (%)				
Grade 1	1.23 (0.51–2.95)	.632	1.11 (0.42–2.92)	.819
Grade 2	1.15 (0.43–3.07)	.772	1.17 (0.43–3.19)	.751
Grade 3	1.08 (0.34–3.39)	.885	0.97 (0.30–3.12)	.964
With CMBs (%)	2.17 (0.89–5.26)	.085	2.76 (1.03–7.38)	.043
1	1.23 (0.40–3.80)	.707	1.19 (0.32–4.41)	.788
2–4	1.40 (0.53–3.67)	.494	1.94 (0.66–5.67)	.222
≥5	2.56 (0.89–7.39)	.081	4.78 (1.18–19.28)	.028
Strictly deep	0.81 (0.24–2.64)	.721	0.97 (0.24–3.83)	.973
Strictly lobar	0.94 (0.29–3.01)	.921	0.85 (0.25–2.86)	.793
Strictly infratentorial	1.21 (0.27–5.44)	.802	1.41 (0.28–7.16)	.674
Mixed location	0.97 (0.34–2.75)	.965	0.86 (0.29–2.53)	.791
With enlarged basal ganglia PVSs (%)				
<10	1.28 (0.62–1.74)	.329	1.56 (0.87–3.11)	.096
10–20	1.68 (0.84–3.36)	.142	1.98 (0.93–4.21)	.076
>20	1.10 (0.42–2.85)	.840	1.16 (0.44–3.07)	.752

Abbreviations: ALIs, asymptomatic lacunar infarctions; CMBs, cerebral microbleeds; CSVD, cerebral small vessel disease; NHISS, the NIH Stroke Scale; PVS, perivascular spaces; WMHs, white matter hyperintensities.

* Adjusted for age, gender, education, admission NIHSS, obesity, hyperlipidemia, history of hypertension, diabetes, coronary heart disease, drinking, smoking.

## 4 DISCUSSION

In our study, we quantified the overall burden of CSVD, as well as individual CSVD markers, and studied the association with cognitive function in 179 newly diagnosed neurosyphilis patients presenting with minor acute ischemic stroke. We found the CSVD loads were greater in neurosyphilis participants compared with syphilis‐negative controls and that was associated with cognitive decline. Longitudinal studies following cognitive function over 12 months indicated that the CSVD burden did impact the long‐term post‐stroke cognitive outcome of neurosyphilis patients.

The CSVD lesions are often seen in the general population, with a strong association with cerebrovascular disease. Although the underlying CSVD pathogenesis is not well understood and may be multifactorial, arteriolosclerosis is also known as a common CSVD type strongly associated with age and hypertension (Wardlaw et al., 2013; Pantoni, 2010). There is increasing evidence that CSVD plays a crucial role in the pathogenesis of post‐stroke cognitive impairment. All the markers of CSVD, including WMHs, CMBs, ALIs, and EPVS, have an independent or combined effect on post‐stroke cognitive impairment (Teng et al., 2017). Recent MRI findings in neurosyphilis were not highly specific, most prominent presenting with white matter lesions in multiple arterial distributions, atrophy, and cerebral infarction (Mehrabian et al., 2012). There are few imaging studies of CSVD in the neurosyphilis population and little evidence for a difference between the prevalence of CSVD in neurosyphilis patients and seronegative controls. Our findings indicated the CSVD prevalence in the neurosyphilis‐related stroke population was increased compared with nonsyphilis patients, accompanied by no increase in atherosclerosis risk factors and even lower age and hypertension rate. It is possible that neurosyphilis is an independent risk factor associated with CSVD, stroke patients with neurosyphilis experience aggravated process of cerebrovascular aging or faster progression of CSVD. Recent extensive evidence strongly suggests that CSVD development may result from chronic immune activation, altered immune homeostasis, and neurovascular dysfunction in the central nervous system (Shoamanesh et al., 2015). An inflammatory and immunological process may play a role in the pathogenesis of CSVD associated with neurosyphilis (Shoamanesh et al., 2015; Rouhl et al., 2012). The medium and small vessels are usually involved in syphilitic vasculitis, with inflammatory changes in the adventitia, along with the fibroblastic proliferation of the media and intima, leading to ischemia and subsequent infarction. Our previous work has established the high incidence of middle cerebral artery stenosis in syphilis‐positive ischemic stroke (Xiang et al., 2021). The growing proportion of small vessel cerebrovascular disease is also an important area for the clinical research agenda based on our findings.

Cerebral microbleeds, one of the key quantifiable neuroimaging features of CSVD, are reported to be highly prevalent in patients with cognitive impairment. Moreover, some studies have previously shown that CMBs, especially in large numbers, are associated with worse performance on tests that measure cognitive function in patients with essential hypertension (Poels et al., 2012; Zhang et al., 2018), ischemic stroke, or TIA (Wang et al., 2015). An accumulating amount of evidence suggests that the WMHs on MRI predicts an increased risk of cognitive decline in the elderly (Wardlaw et al., 2013; Zamboni et al., 2019). However, there was still a lack of evidence for CMBs and WMHs as correlates of neurosyphilis‐associated cognitive impairment. We conducted this one‐year follow‐up study in neurosyphilis presenting with minor ischemic stroke. We demonstrated that cognitive decline strongly correlated with CMBs and WMHs, and then found that CMBs can predict post‐stroke cognitive recovery. Lacunars, a representative marker of CSVD, is often mentioned with future cognitive decline in the general population (Benjamin et al., 2018), whereas it was not a predictor of post‐stroke cognitive outcome in our findings. Furthermore, baseline EPVs scores showed no association with cognitive function. Mechanisms by which CMBs and WMHs influence cognitive function remain speculative. Histopathologic studies have shown that CMBs may disrupt the microstructure of the surrounding white matter tracts, leading to neural network damage superimposed by the effects of frequently co‐occurring WMHs and ALIs involved in cognitive decline (Pasi et al., 2017). It is possible that the small vessel event triggers and aggregates cognitive impairment processes through multiple mechanisms such as enhanced inflammatory response or ischemic compensatory processes. These MRI markers do not occur separately, investigating the effect of the combination and accumulating of these individual markers on cognitive function is more valuable.

In the elderly, the total burden of CSVD is a leading cause of the cognitive decline and predicts future risk of stroke and dementia (Lau et al., 2017; Uiterwijk et al., 2016). Although all the markers of CSVD are individually associated with cognitive decline, the combined effect of these markers possibly provides more useful information on cognitive function. It is recognized that cognitive impairment is not only common in general paresis, but also highly prevalent in meningovascular neurosyphilis with a slow and insidious course of cognitive decline. Accumulation of CSVD is probably the main mechanism linking vascular risk factors to cognitive decline. An association between neurosyphilis and CSVD would be of major importance to those patients living with syphilis because of the increased risk of stroke and dementia. We reported the novel finding that the total CSVD burden is associated with decreased MoCA performance, implying that accumulating brain damage is accompanied by reduced cognitive function in neurosyphilis.

Post‐stroke dementia is common but has complicated mechanisms that are not fully understood. Cognitive performance in acute stroke might not represent later cognition, as performance might improve over longer time periods. There has been a recent interest in identifying the improvements and demonstrating their association with CSVD (Mok et al., 2017). We use MoCA, a well‐validated tool with high sensitivity and specificity for mild cognitive impairment arising from vascular factors to screen cognitive decline and estimate cognitive amelioration in post‐stroke patients with or without neurosyphilis. Although the underlying mechanisms of cognitive impairment in neurosyphilis are heterogeneous, we raise the intriguing possibility that CSVD accounts for some early cognitive changes in the neurosyphilis population presenting with ischemic stroke. Ischemic stroke could have a negative impact on cognitive function, although our results indicate that the baseline CSVD may be a more important determinant of cognitive function in neurosyphilis patients. The ability to recover from post‐stroke cognitive decline is related to the integrity of neural networks, which are disrupted in the context of extensive CSVD, thus impairing the neural plasticity and compensatory mechanisms. Neurosyphilis patients with a higher CSVD burden are therefore more predisposed to cognitive decline and are less likely to recover from the stroke. Therefore, reduction in CSVD risk in neurosyphilis has been supposed to prevent further decline in cognition. Syphilis, one of the modifiable CSVD factors identified herein, along with other vascular risks such as hypertension, should be treated aggressively to prevent deterioration of cognitive function and/or underlying CSVD in these individuals. Meanwhile, our findings highlight the importance of routine monitoring of syphilis serology in stroke patients.

Our study is limited by the following aspects. Firstly, our study focused on a specific demographic group recruited from the same stroke center and limited to minor ischemic stroke patients without a history of dementia, which may limit the generalizability of our findings to other syphilis infected populations. Future research should include these participants and larger sample sizes. Additionally, the construction of the CSVD scale has some intrinsic limitations, which do not take into account all aspects, like the extent or location of each marker. In our study, ALIs was not a predictor of post‐stroke cognitive outcome. A possible explanation is that we did not integrate the number of lacunes, although it was previously found that a larger number of lacunes is associated with decreased cognitive performance in the elderly (Aggarwal et al., 2012). Finally, we only employed a single measure (the MoCA) to estimate cognitive performance at acute and 12‐month follow‐up time points. MoCA is primarily a screening tool that is likely to underestimate the severity and breadth of cognitive impairment. Assessment at additional time points, both before and beyond 12 months (for example, at 3 or 6 months, 2 years), and using a comprehensive neuropsychological test battery would allow us to better estimate post‐stroke cognitive development.

## 5 CONCLUSION

In conclusion, we provided insight into the impact that CSVD has on cognitive impairment in neurosyphilis patients presenting with ischemic stroke. CSVD is more common in neurosyphilis participants compared with syphilis‐negative controls and that was associated with cognitive decline. CSVD is possible to be a reliable marker to predict the cognitive outcome in stroke patients with neurosyphilis.

## CONFLICT OF INTEREST

The authors declare that there is no conflict of interest.

## AUTHOR CONTRIBUTIONS

Wei Yue and Shuping Hou contributed to the concept initiation and study design. Lei Xiang and Tao Zhang contributed to data analysis and manuscript drafting. Biao Zhang and Chao Zhang contributed to data acquisition.

### PEER REVIEW

The peer review history for this article is available at https://publons.com/publon/10.1002/brb3.2187.

## Data Availability

The data that support the findings of this study are available on request from the corresponding author. The data are not publicly available due to privacy or ethical restrictions.
